# Development of an Indirect ELISA Based on Spike Protein to Detect Antibodies against Feline Coronavirus

**DOI:** 10.3390/v13122496

**Published:** 2021-12-13

**Authors:** Bo Dong, Gaoqiang Zhang, Xiaodong Zhang, Xufei Chen, Meiling Zhang, Linglin Li, Weiming Lin

**Affiliations:** 1 Department of Veterinary Medicine and Animal Science, College of Life Science of Longyan University, Longyan 364012, China; Y2019004@lyun.edu.cn (G.Z.); Y202003@lyun.edu.cn (X.Z.); 2018082707@lyun.edu.cn (X.C.); 2019085641@lyun.edu.cn (M.Z.); 2019085612@lyun.edu.cn (L.L.); 2 Fujian Provincial Key Laboratory for the Prevention and Control of Animal Infectious Diseases and Biotechnology, Longyan 364012, China

**Keywords:** feline coronaviruses, spike protein, ELISA, diagnosis, serum epidemiology

## Abstract

Feline coronavirus (FCoV) is a pathogenic virus commonly found in cats that causes a benign enteric illness and fatal systemic disease, feline infectious peritonitis. The development of serological diagnostic tools for FCoV is helpful for clinical diagnosis and epidemiological investigation. Therefore, this study aimed to develop an indirect enzyme-linked immunosorbent assay (iELISA) to detect antibodies against FCoV using histidine-tagged recombinant spike protein. FCoV S protein (1127–1400 aa) was expressed and used as an antigen to establish an ELISA. Mice and rabbits immunized with the protein produced antibodies that were recognized and bound to the protein. The intra-assay coefficient of variation (CV) was 1.15–5.04% and the inter-assay CV was 4.28–15.13%, suggesting an acceptable repeatability. iELISA did not cross-react with antisera against other feline viruses. The receiver operating characteristic curve analysis revealed an 86.7% sensitivity and 93.3% specificity for iELISA. Serum samples (n = 107) were tested for anti-FCoV antibodies, and 70.09% of samples were positive for antibodies against FCoV. The iELISA developed in our study can be used to measure serum FCoV antibodies due to its acceptable repeatability, sensitivity, and specificity. Additionally, field sample analysis data demonstrated that FCoV is highly prevalent in cat populations in Fujian province, China.

## 1. Introduction

Feline coronavirus (FCoV) is a positive-stranded RNA virus that belongs to the family *Coronaviridae* and genus *Alphacoronavirus*, which is frequently found in cats [1]. FCoV exhibits two pathogenic forms: one causes subclinical or mild intestinal infections and the other causes fatal feline infectious peritonitis (FIP). The low-virulence form is called feline enteric coronavirus (FECV), while the high-virulence form is called feline infectious peritonitis virus (FIPV) [2]. FIPV causes fatal, immune-mediated, suppurative granulomatous disease. FIPV infections have a high fatality rate, and infected cats die within a short time [3]. FIPV has enhanced monocyte/macrophage tropism, which is reflected in the continuous monocyte replication and subsequent activation. These activated monocytes carry the virus in the blood and, due to complex interactions with endothelial cells, lead to granulomatous phlebitis, a hallmark of FIP [4].

Similar to other coronaviruses, the FCoV genome has 11 open reading frames (ORFs) that encode four structural proteins (spike (S), envelope (E), membrane (M), and nucleocapsid (N)) and seven nonstructural proteins: accessory proteins 3a, 3b, 3c, 7a, and 7b and replicases 1a and 1b [5]. As the largest structural protein on the surface of the virus, the S protein is a major viral regulator in host cell entry [6]. The S protein mediates the binding of the virus to the host cells via its receptor and membrane fusion during the process of invasion [7]. Studies have shown that the S protein plays a crucial biological role in the process of invading host cells through membrane fusion after the binding of virions to cell surface receptors [8]. In addition, the S protein contains antigenic epitopes that mediate the production of antibodies in infected hosts and play an important role in antigenic recognition [9]. Therefore, the S protein is one of the most important target proteins in research on the genetic engineering of vaccines and diagnostic technology.

According to its serological characteristics, FCoV can be divided into type I and type II, and both serotypes exist in FECV and FIPV [10]. This antigenic classification is based on differences in the amino acid sequence of the FCoV S protein and antibody neutralization [11]. Type I FCoV is the primary serotype, and its S protein is completely derived from FCoV. Viral strains from this serotype are clinically more common. Type II FCoV is less common than type I FCoV and is derived from a double recombination between FCoV and canine coronavirus (CCoV). Amino acid sequence homology analysis has revealed that type II FCoV S protein showed a significantly higher homology with the S protein of type II CCoV or transmissible gastroenteritis virus (TEGV) than with the S protein of type I FCoV [10]. In addition to showing significant serological differences, type I and type II FCoV are considered to have distinct taxonomic characteristics consistent with their different biological characteristics. These characteristics also exist in type I and type II CCoV. The genus Alphacoronavirus has two independent branches: one clade includes type I FCoV and CCoV, while the other clade includes type II FCoV and CCoV and TGEV-like viruses. Therefore, it has also been proposed to classify these into two separate clades within the genus Alphacoronavirus [12]. Of the two serotypes, type I FCoV infection shows a high prevalence of 80–95% in Europe and the United States, while type II FCoV has a low prevalence [13,14]. In addition, type I FCoV strains have high prevalence in both FIP-infected cats and clinically healthy cats in China [15,16].

FCoV antibodies can be used to screen for FCoV infection before entering a cattery or other FCoV-free households to determine the efficacy of early weaning and isolation [17,18], although the latter are rarely done. Anti-FCoV antibody measurement may be more useful than virus detection because antibody titers below 1:10 indicate that cats are unlikely to be shedding FCoV and that detection of FcoV would be difficult even when using quantificational real-time polymerase chain reaction (RT-PCR) [19]. In addition, cats infected with FIPV usually have high antibody titers against FCoV [20].

Compared with antibody detection methods based on whole virus, recombinant protein expressed and purified by *Escherichia coli* and used for indirect ELISA can avoid the occurrence of animal-derived cross-reactivity and reduce false positives [21]. In the present study, a partially truncated S protein was selected as the coating antigen for the first time to develop an indirect ELISA to detect anti-FCoV antibodies. Furthermore, we validated the receiver operating characteristic (ROC) curve, sensitivity, and repeatability of the iELISA. This study aimed to provide a potential serological diagnostic tool for FCoV infection.

## 2. Materials and Methods

### 2.1. Animal and Serum Samples and Antibodies

Six-week-old BALB/c female mice weighing 20–25 g and a female New Zealand White rabbit procured from Wu’s Experimental Animal Trading Co., Ltd. (Fujian, China), were housed under standard and ventilated conditions in the animal care facility of Longyan University. Antisera against coronavirus, feline panleukopenia virus (FPV), feline calicivirus (FCV), and feline herpesvirus (FHV) were obtained from naturally infected domestic cats and the Animal Hospital of Longyan University. Monoclonal antibodies against histidine (His) were obtained from TransGen Biotech Co., Ltd. (Beijing, China). A serum sample named FJLY20201, which was collected from one cat diagnosed by the animal hospital as being positive for FCoV infection and found, by Western blot, to react specifically with FCoV-SP that was selected fragment in this study, was used as a positive control (P). A FJLY05 sample which was negative for FCoV infection was used as the negative control (N). Additionally, 30 negative samples and 30 positive samples were collected from uninfected or infected cats respectively for assessment of the diagnostic sensitivity and specificity. And 55 samples detected negative by western blot and iELISA were used for determine the cut-off value. A total of 107 cat serum samples were collected from Fuzhou, Xiamen, and Longyan in Fujian Province of China. The serum samples were used after obtaining ethical approval from the Committee on the Ethics of Animal Experiments of Longyan University (20201101A, November 2020). The study was conducted in compliance with the ARRIVE guidelines. This study was performed in accordance with the National Guidelines for the Care and Use of Laboratory Animals (CNAS-CL06, 2018). Informed consent was obtained from the cats’ owners prior to sample collection. Sampling and data publication were approved by the cats’ owners.

### 2.2. Antigen Selection and Vector Construction

The nucleotide sequence of the entire S gene of FCoV was obtained from the GenBank database at the National Center for Biotechnology Information (NCBI) website (accession no. EU186072). The S protein was analyzed using the Editseq software from DNAStar package software, and epitopes were predicted and easily expressed fragments were selected. The selected fragment was named FCoV-SP, and the target gene was synthesized by referring to published strain sequences from GenBank. The recombinant expression vector, pET-28a-SP, was obtained from Shanghai Sangon Biological Engineering Technology and Services Co., Ltd. (Shanghai, China).

### 2.3. Expression of Recombinant FCoV-SP Protein

Recombinant plasmids were transformed into *E. coli* BL21 (DE3) cells, and FCoV-SP gene expression was induced using isopropyl β-D-1-thiogalactopyranoside (IPTG) at a final concentration of 1.0 mM at 37 °C for 4 h. Protein expression was analyzed using 12% sodium dodecyl sulfate-polyacrylamide gel electrophoresis (SDS-PAGE). Moreover, recombinant FCoV-SP proteins were purified with an Ni-NT affinity chromatography column based on a previous study [22] and stored at −80 °C for future use.

### 2.4. Western Blotting of the FCoV-SP Protein

Purified FCoV-SP proteins with a His-taq were subjected to 12% SDS-PAGE and transferred to a polyvinylidene fluoride (PVDF) membrane using a semi-dry transfer apparatus (Bio-Rad, Hercules, CA, USA). The recombinant protein was detected and a predicted molecular weight of 32 kDa was confirmed by Western blotting using a 6X His mAb (TransGen Biotech, Beijing, China).

### 2.5. Immunogenicity Assessment

BALB/c mice were subcutaneously injected with purified FCoV-SP protein (50 µg/mouse) emulsified using Freund’s complete adjuvant. One week later, mice were injected with a mixture of the same antigen and Freund’s complete adjuvant. This was followed by weekly injections for 3 weeks. The same procedure was followed in rabbits but with 200 µg/rabbit of FCoV-SP. Caudal artery blood or auricular venous blood was collected and serum was separated and stored at −80 °C. After the last blood collection, animals from each group were euthanized in a CO_2_ chamber. The antibody titer was determined using the iELISA developed in the present study. Sera were serially diluted from 1:200 to 1:102,400 in PBS to detect antibody titration.

### 2.6. iELISA Procedures for Detecting Antibodies against FCoV

The conditions of the iELISA, including the concentrations of coated antigen, blocking solution, sera, and HRP-conjugated goat anti-cat IgG and their incubation times, were optimized according to the P/N value. The best reaction conditions were as follows: ELISA plate (Costa, Corning, NY, USA) wells were coated with 4 μg/mL of purified His-tagged FCoV-SP protein in 0.05 mol/L of carbonate buffer (pH 9.6) and incubated overnight at 4 °C. After washing with phosphate-buffered saline tween (PBST) three times, 100 µL of 5% skimmed milk was added to each well and incubated at 37 °C for 1 h. After washing with PBST three times, 50 µL of serum at a dilution of 1:400 was added to the wells and incubated at 37 °C for 1.5 h. After washing with PBST three times, HRP-conjugated goat anti-cat IgG diluted 1:20,000 in PBST was added and allowed to incubate at room temperature (RT) for 1 h. After adding 100 µL of 3,3,5,5-tetramethylbenzidine (TMB) substrate solution and incubating at RT for 6 min, the reaction was stopped by adding 100 µL of ELISA Stop Solution (Solarbio, Beijing, China), and the OD_450_ was measured.

### 2.7. Determination of the Cut-Off Value

The result of each sample percentage reactivity (PR) value is in the conversion, according to the following formula: PR value = [(the|OD value of the tested sample—the negative control)|/(the OD value of the positive control—the negative control)] × 100%. The 55 FCoV-negative serum samples were detected by iELISA. The mean OD value of each sample was analyzed after three tests. The PR cut-off value for the serum-based SP ELISA was calculated using the mean PR value (X) of 55 FCoV-negative serum samples plus two standard deviations (SDs). When the PR value of the sample to be tested was greater than or equal to the cut-off value, it was determined to be positive. If not, it was negative.

### 2.8. Check for the Coefficient of Variation (CV)

Three positive serum samples and two negative serum samples were selected to evaluate intra-assay variation and inter-assay variation. The value of each determination was expressed as the mean, standard deviation, and coefficient of variation (CV%) of repeated measurements. The intra-assay CVs were analyzed for each sample in triplicate within the same assay, and the inter-assay CVs were evaluated for each sample on five different days within 1 week.

### 2.9. Specificity Test

To evaluate the specificity of the iELISA, antisera against FPV, FCV, and FHV were screened. The reaction conditions were the same as those used for the iELISA. Each sample was tested three times, and the PR value was used to determine whether the sample was positive or negative.

### 2.10. Assessment of the Diagnostic Sensitivity and Specificity

The diagnostic sensitivity and specificity were evaluated by receiver operator characteristic (ROC) analysis using 30 FCoV-positive serum samples and 30 negative samples, as well as the cumulative data from all samples using Western blotting as the reference method for sample classification. Statistical analysis was performed using the SPSS software (Version 11.5 for Windows, SPSS Inc., Chicago, IL, USA).

### 2.11. Detection of FCoV in Field Samples

A total of 107 serum samples were collected from Fuzhou, Xiamen, and Longyan in Fujian Province of China. All serum samples were tested using the iELISA kit. The mean PR value was used to determine whether the sample was positive or negative.

### 2.12. Statistical Analysis

A chi-square test was used to compare the prevalence rates between the two groups of different ages and genders. Statistical analyses were performed using the SPSS software (version 19.0; SPSS, Inc., Chicago, IL, USA). Statistical significance was set at *p* < 0.05. All data were visualized using the GraphPad Prism 8.0 software (GraphPad Software, San Diego, CA, USA).

## 3. Results

### 3.1. Production of the FCoV-SP Protein

**Figure 1:**
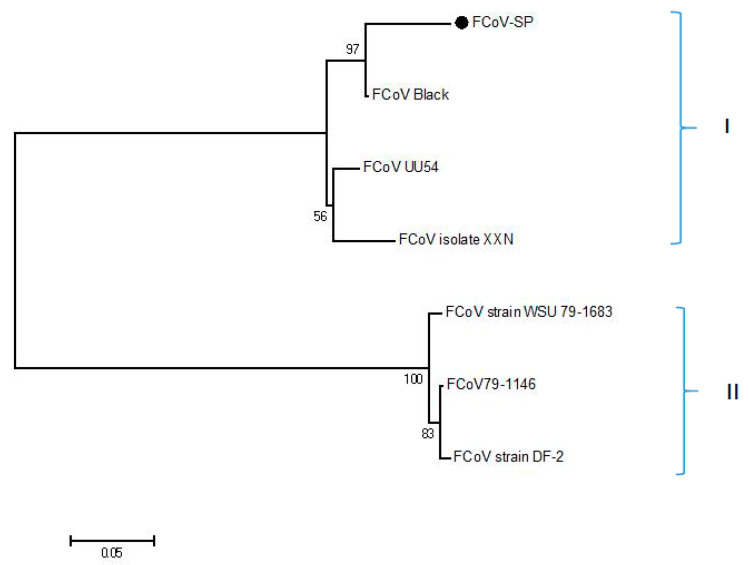
Phylogenetic analysis. Phylogenetic tree derived from amino acid sequences was constructed by MEGA version 5.2 using the neighbor-joining method with the p-distance model, 1000 bootstrap replicates. ● represents FCoV-SP identified in our study. I: type I, II: type II.

**Figure 2:**
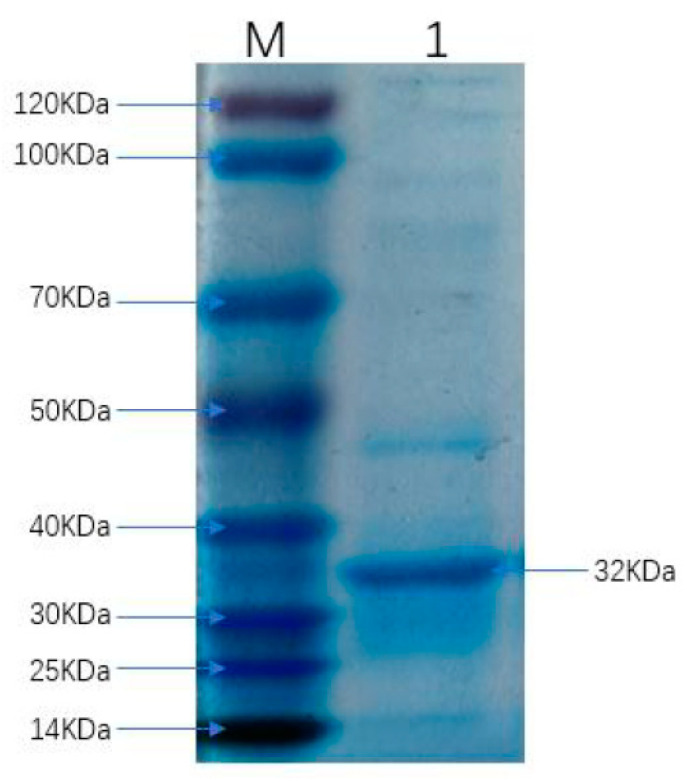
SDS-PAGE analysis of the purification of FCoV-SP. Lane M: protein marker (14 kDa–120 kDa); Lane 1: FCoV-SP protein.

**Figure 3:**
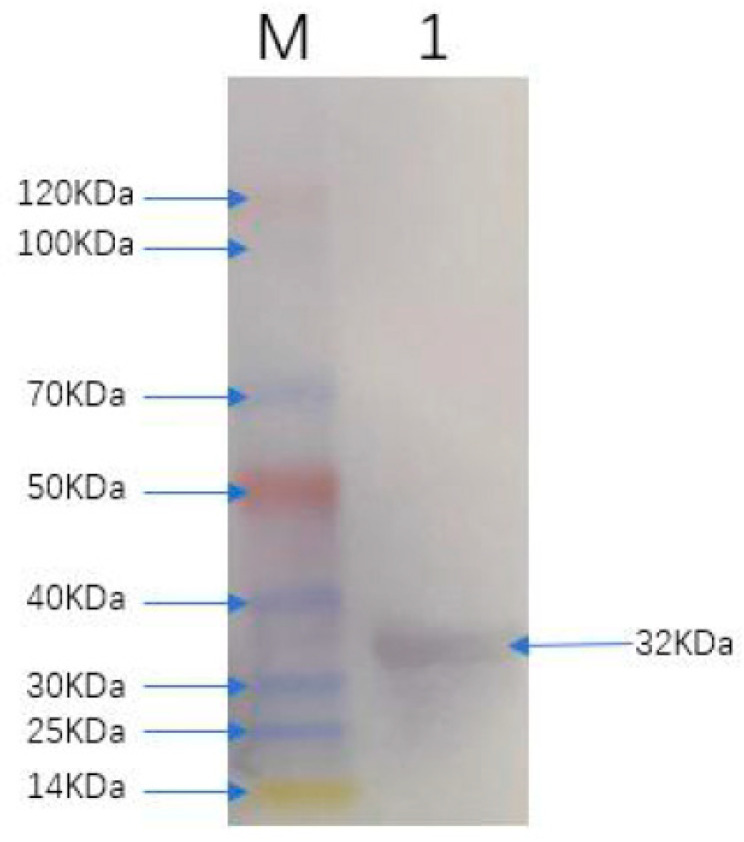
Western blot analysis of FCoV-SP. Lane M: protein marker (14 kDa–120 kDa); Lane 1: the purified FCoV-SP protein.

By DNAstar analysis, the region spanning amino acids 1127–1400 with B cell epitopes and exposure in the C-terminal of FCoV S protein was selected in this study (Supplementary Materials), and this was named as FCoV-SP. Multi-sequence alignment analysis of amino acid sequences revealed that the identity between FCoV-SP and other type I strains in our study was 93.1–100.0%, while the identity between FCoV-SP and other type II FCoV strains was 62.9–63.2%. The phylogenetic analysis of FCoV-SP showed that it was included in the type I FCoV clade (Figure 1). FCoV-SP was expressed in the *E. coli* BL21 (DE3) strain using the vector pET-28a. The results showed that the recombinant protein was successfully expressed and purified at approximately 32 kDa (Figure 2). In addition, FCoV-SP was verified by Western blotting using the anti-His-tag monoclonal antibody as the primary antibody (Figure 3).

### 3.2. Immunogenicity Evaluation of FCoV-SP Protein

**Figure 4:**
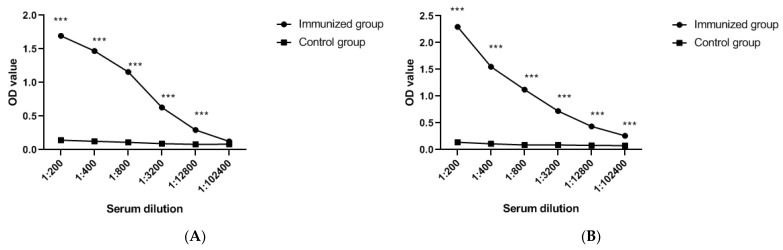
(**A**) Mice and (**B**) rabbit antibody titers against FCoV-SP after three immunizations.Means were analyzed using GraphPad Prism software version 8.0 for Windows (San Diego, CA, USA). The blue dashed line indicates the PR value (30.83). *** *p* < 0.05. OD: optical density.

In serum tests using the established indirect ELISA, the titers of antibodies in mice and rabbits after the third round of enhanced immunity were different from those in the controls (*p* < 0.05) (Figure 4A,B).

### 3.3. Cut-Off Value of the iELISA

**Figure 5:**
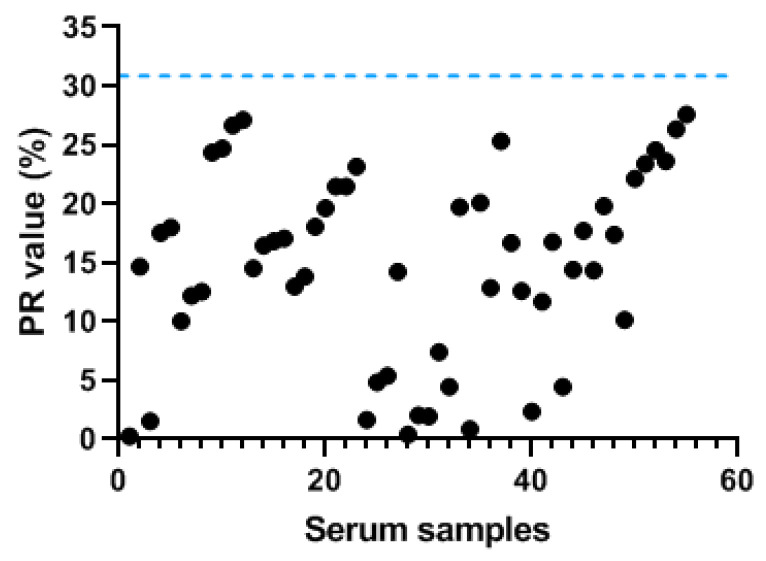
Determination of the cut-off value of the iELISA. The blue dashed line indicates the PR value (30.83).

The 55 negative serum samples were tested to determine the cut-off value of the iELISA. The average PR value of negative samples was statistically analyzed and calculated as 14.75 and the standard deviation (SD) was 8.04. The cut-off value was determined as the mean value of the negative serum + 2 × SD. All PR values of the serum samples > 30.83 were determined to be positive. If not, the serum was considered to be negative (Figure 5).

### 3.4. Repeatability of the iELISA

To assess the repeatability of the iELISA, five samples were selected. The results revealed that the intra-assay CVs ranged from 1.15% to 5.04%, while the inter-assay CVs ranged from 4.28% to 15.13% (Table 1). In general, all CV% values were <20%, indicating the good repeatability of iELISA.

### 3.5. Specificity Assessment

**Figure 6:**
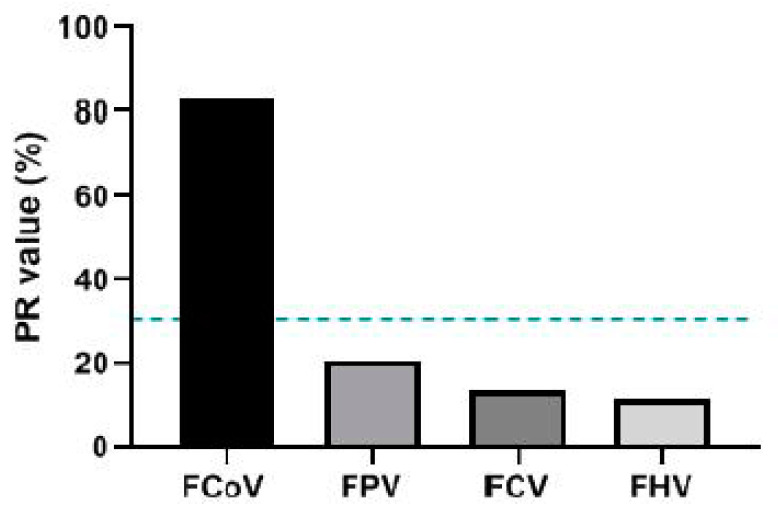
Specificity test of the iELISA. The blue dashed line indicates the PR value (30.83). FPV: feline panleukopenia virus, FCV: feline calicivirus, FHV: feline herpesvirus, PR: percentage reactivity.

Antisera against FPV, FCV, and FHV were used to test the specificity of iELISA. The results showed that iELISA did not cross-react with these antisera (Figure 6).

### 3.6. Diagnostic Sensitivity and Specificity of iELISA

**Figure 7:**
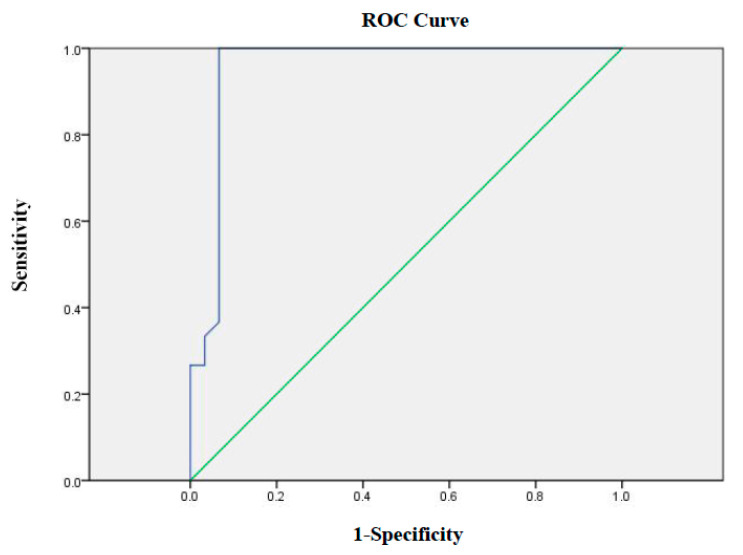
The ROC curve using Western blotting as a diagnostic standard.

Sixty samples were tested by Western blotting and iELISA. For the positive samples, 30 out of 30 samples were correctly identified by the test. Of the 30 negative samples, 28 were correctly identified using the test (Table 2). The ROC curve showed a sensitivity of 86.7%, a specificity of 93.3%, and an area under the ROC curve (ROC AUC) of 0.954 (Figure 7).

### 3.7. Antibody Identification in Field Samples

**Figure 8:**
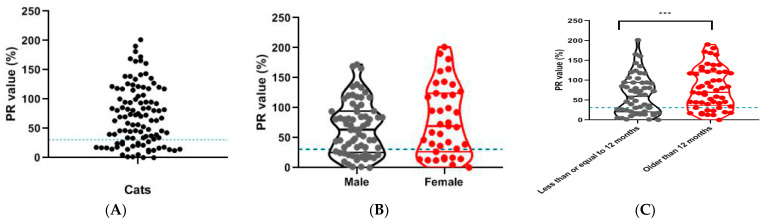
Field serum samples underwent anti-FCoV antibody testing. (**A**) In total, 107 serum samples were analysed. A total of 75 samples were antibody positive. (**B**) Cats of different genders were analysed. Of all cats, 46 (67.64%) males were antibody positive and 29 (74.36%) females were antibody positive. *p* > 0.05. (**C**) Cats of different ages were analysed. Of all cats, 34 (62.96%) cats of 12 months of age or younger and 41 (77.36%) cats older than 12 months were antibody positive. Violin plots, medians, and quartiles were analyzed using GraphPad Prism software version 8.0 for Windows (San Diego, CA, USA). The blue dashed line indicates the PR value (30.83). *** *p* < 0.05.

A total of 107 field feline serum samples were used to detect the antibodies. The results showed that 70.09% of the samples (75/107) were positive for antibodies against FCoV (Figure 8A). Among males, 67.64% of samples (46/68) were positive for antibodies against FCoV; among females, 74.36% of samples (29/39) were positive for antibodies against FCoV (Figure 8B). Among the 107 felines, 54 were ≤12 months old, and 34 (62.96%) of these showed positive results. Of the 53 felines aged over 12 months, 41 (77.36%) had positive results (Figure 8C).

## 4. Discussion

In this study, we developed an iELISA to detect antibodies against FCoV. iELISAs based on cell culture virus preparation could limit large-scale detection and lead to a low specificity and high background interference [23]. In addition, among the viral structural proteins, S protein could be considered the most important in terms of FCoV pathogenesis because it mediates receptor binding and virus–cell membrane fusion and is rich in antigenic and neutralizing epitopes [24]. In other coronaviruses, such as porcine epidemic diarrhea virus (PEDV), the S protein iELISA is more sensitive and specific and has no cross-reactivity with other viruses [25]. Therefore, in this study, the FCoV S protein was selected for prokaryotic expression in *E. coli*. We first used bioinformatic techniques to analyze the accessibility region of the S protein. Then, we selected a region suitable for prokaryotic expression. SDS-PAGE and Western blot results showed that the expression and purification of the recombinant protein were as effective as expected. Moreover, immunogenicity analysis showed that the antibodies produced by injecting BALB/c mice could effectively recognize and bind to recombinant proteins. In addition, specificity tests indicated that no cross-reactivity with FPV, FCV, or FHV was detected with the iELISA developed in the present study.

An ideal test should have a good repeatability, have a high sensitivity and specificity, provide results with small sample quantities, and provide quantitative values [26]. The repeatability results showed that intra -and inter-assay CVs were <20%, which is acceptable [27]; thus, these results indicate acceptable repeatability estimates for this iELISA [28]. Additionally, in this study, Western blotting was used to validate the diagnostic efficacy of ELISA. The ROC curve was used to evaluate the iELISA, and the results showed that the iELISA exhibited a relative sensitivity of 86.7%, a relative specificity of 93.3%, and an ROC AUC of 0.954, suggesting that the iELISA developed in the present study has potential application as a diagnostic antibody test.

Since there is currently no FCoV vaccine available in China, the detection of antibodies can help in the diagnosis of FCoV infection and help determine prevalence. FCoV infection is often found in cats, with antibodies reported in 80–90% of cats in a cattery and in 10–50% of domestic cats [21]. In the present study, we collected 107 serum samples from animal hospitals in Fujian Province from domestic cats. Antibody screening using the iELISA showed a 70.09% positive rate, highlighting the high FCoV prevalence in cats. In addition, the results showed that the prevalence of FCoV was not significantly different between male and female cats, indicating that FCoV infection was independent of sex in this study. Cats of all ages can be infected with FCoV, although young animals are said to be more susceptible to coronavirus infection [29,30]. However, the results of this study showed that the prevalence of FCoV was significantly higher in cats greater than 12 months old; the results of this study need to be further analyzed by antibody-screening studies with larger sample sizes in the future.

Of the 107 samples in this study, six were from confirmed FIPV-infected cats (data not presented). The iELISA results showed that all six samples were positive, indicating that the iELISA developed in this study showed evidence of FCoV infection in these FIPV-infected cats. Moreover, 3 of the 107 samples were obtained from treated cats with a history of FIPV. All three cats were diagnosed and treated for FIPV infection, and serum samples were collected one month after treatment. The iELISA results showed that all of them were negative. This may be due to the decrease in antibody titer in these three cats after effective treatment, resulting in negative results. Additionally, the results of two serum samples from cats with suspected FIPV infection were positive. FIPV infection was confirmed by immunohistochemistry in both cats, indicating that the iELISA in this study can be used as a diagnostic method to help determine the presence of FCoV infection in cats.

This study has the following limitations. In general, a novel ELISA-based diagnostic method needs to be validated by comparison with the same or similar commercial ELISA kits or antibody assays that do not use the same purified protein. However, the iELISA established in this study was validated by comparing the Western blotting results for FCoV-SP as antigen with the iELISA results, and the relative specificity and relative sensitivity were analyzed by an ROC curve. The results showed that the iELISA established in this study has potential application as a diagnostic antibody test. However, further comparative analysis of this iELISA is needed. In addition, none of the antiserum samples collected in this study were from identified type II specific antisera. Although sequence analysis showed a low similarity between FCoV-SP and type II FCoV sequences, further verification is needed to determine whether the iELISA will also detect type II-specific samples or might effectively distinguish between type I and II samples. Moreover, maternal antibodies may affect serological testing in kittens. The presence of maternal antibodies in the younger cats (12 months or less of age; the youngest cats were 2 months old) in this study also requires further verification.

## 5. Conclusions

The developed iELISA has acceptable repeatability and specificity for the detection of anti-FCoV antibodies in serum and has the potential for use in the epidemiological investigation and the serological diagnosis of FCoV infection. In addition, the results have shown a high prevalence in limited serological investigations in Fujian province, China; therefore, an extensive serological investigation of the epidemiology of FCoV in China should be performed in future studies.

## Figures and Tables

**Table 1:** Estimates of coefficients of variation (CV) from 5 samples.

	Sample	X	SD	CV%
Intra-assay	1	2.03	0.03	1.63%
	2	2.26	0.03	1.15%
	3	1.99	0.05	2.40%
	4	0.14	0.01	4.13%
	5	0.19	0.01	5.04%
Inter-assay	1	2.31	0.22	9.28%
	2	2.21	0.14	6.09%
	3	2.07	0.09	4.28%
	4	0.16	0.02	15.13%
	5	0.21	0.01	6.80%

**Table 2:** Results for serum samples that were tested to compare between Western blot and iELISA.

iELISA	Result of Western Blot		Total
	Positive	Negative	
Positive	30	2	32
Negative	0	28	28

## Data Availability

The data analyzed during the current study are available from the corresponding author upon reasonable request.

## Supplementary Materials

The following are available online at https://www.mdpi.com/article/10.3390/v13122496/s1. Figure S1: Bioinformatics analysis diagram of FCoV S protein.
