# Supplementary material for: Development of an Indirect ELISA Based on Spike Protein to Detect Antibodies against Feline Coronavirus
Source: Viruses. 2021 Dec 13;13(12):2496. doi: 10.3390/v13122496 (PMC8707903; doi:10.3390/v13122496)
Supplement: Supplementary file 1 [file viruses-13-02496-s001.zip › viruses-1438787-supplementary.pdf]

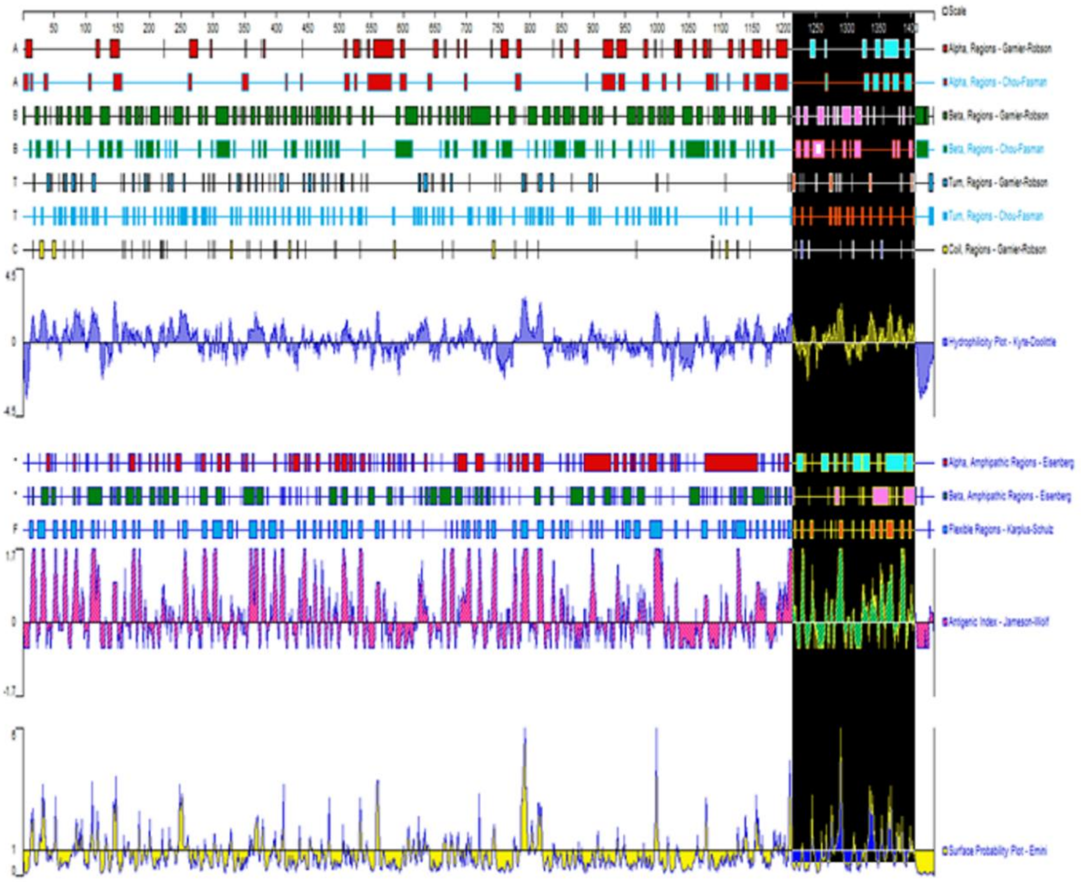

Figure S1. Bioinformatics analysis diagram of FCoV S protein
The dark areas are selected for expression.
